# ManyZoos: A New Collaborative Approach to Multi‐Institution Research in Zoos

**DOI:** 10.1002/zoo.70017

**Published:** 2025-07-29

**Authors:** Lisa P. Barrett, Fay E. Clark, Marianne S. Freeman, Ellen Williams, Victoria L. O'Connor

**Affiliations:** ^1^ Center for the Integrative Study of Animal Behavior Indiana University Bloomington Bloomington Indiana USA; ^2^ School of Psychological Science University of Bristol Bristol UK; ^3^ Animal Health and Welfare Research Centre University Centre Sparsholt Winchester UK; ^4^ Department of Animal Health, Behaviour & Welfare Animal Behaviour and Welfare Research Group, Animal Sciences Research Centre Harper Adams University Edgmond UK; ^5^ Department of Psychology Oakland University Rochester Michigan USA; ^6^ Bergen County Zoo Van Saun County Park Paramus New Jersey USA

**Keywords:** big data, collaboration, consortium, open science, replicability

## Abstract

Open science and big data approaches (i.e., approaches which enable the development of large and complex data sets) facilitate comparative analyses and thus more robust, evidence‐based decision‐making. Whilst there has been an increase in published research arising from zoological institutions over several decades, most research has arisen from small‐scale case studies, often involving one or two zoos from a small geographical radius. Data from several zoos can be combined and compared retrospectively, but this is difficult when studies adopt different methods. The benefit of wider, simultaneous multi‐institution research was recently demonstrated when researchers assessed the impact of zoo closures during the COVID‐19 pandemic. In this paper, we introduce a new consortium initiative called ManyZoos, which aims to address the critical need for zoo science to expand even further geographically while incorporating additional institutions and disciplines. Like other “Many X” initiatives (e.g., ManyPrimates, ManyDogs), ManyZoos aims to foster more productive research collaborations between zoological collections and other animal collections, academia, government, and nongovernment organizations. In doing so, ManyZoos will address several current limitations of zoo research including small sample sizes and siloed expertise. ManyZoos embeds collaboration at every stage of research, from study conception to dissemination of results, producing large open data sets with transparent protocols. ManyZoos has the potential to lead to more robust, evidence‐based decision‐making for zoo animal management and conservation.

## 1 Introduction

The key goals of zoo research are to enhance animal welfare, conserve species, and evaluate the impact of zoos in society (Barongi et al. 2015; Binding et al. 2020; Spooner et al. 2023). The first “scientific” zoos were established in the 19th century in a handful of major cities (i.e., Paris, London, Vienna, Philadelphia, and New York; Hosey et al. 2013), but it was only during the latter half of the 20th century that research became universally associated with zoos and the field of “zoo biology” was born (Hvilsom et al. 2020; Loh et al. 2018). Now, the European Association of Zoos and Aquaria (EAZA) emphasizes the importance of ethical and effective research to improve decision‐making and management (Reid et al. 2008). The Association of Zoos and Aquariums (AZA) similarly expects member zoos to advance scientific knowledge while enhancing species conservation and educating the public (AZA 2025).

Modern zoos and aquariums are undertaking a wealth of research. The research output of 288 AZA member zoos across a 10‐year period was 5175 publications receiving 81,342 citations (Loh et al. 2018). Similarly, 291 EAZA member zoos produced 3345 publications (45,821 citations) across a 10‐year period (Hvilsom et al. 2020). However, zoo research has come under criticism for low statistical power (i.e., the inability to detect true effects in data samples) and a lack of generalization of results to wider situations, such as an entire species (Alligood et al. 2017; Kuhar 2006). While not all study findings need to be generalized (e.g., case studies on specific welfare issues), many animal husbandry and conservation questions are widespread across the zoo community (Barongi et al. 2015), and there are significant benefits from zoos and researchers working together, rather than isolated studies needing to “reinvent the wheel.” Moving forward, there has been a call for zoos to work at a greater collaborative level (de Figueiredo and Díez‐León 2022; Garcia‐Pelegrin et al. 2022; Gübert et al. 2023; Hopper 2017) with the aim to produce more impactful research.

## 2 Benefits of Multi‐Institution Research

### 2.1 Sample Size and External Validity

Sample size (N) in a single zoo can be small due to restricted enclosure sizes and other difficulties of housing large groups (Kleiman 1994). For example, some species may be kept in dyads based on sex, or single groups (Kuhar 2006). Thus, there is support for single‐N and small‐N zoo research (Saudargas and Drummer 1996) within the zoo community because it is understood there are only finite numbers of rare and inaccessible species with which to work. Small‐N populations can equate to small sample sizes (depending on the focus of study), which may violate statistical assumptions needed for the majority of inferential statistics. Whilst researchers have devised solutions to overcome analytical issues affecting small sample sizes (Hopkin et al. 2015), these may be more complex, or the data may not be suitable for such analyses. Small‐N studies can sometimes be viewed as less robust or impactful (Saudargas and Drummer 1996). The lack of understanding and tolerance in the wider scientific community for small‐N research can restrict opportunities for the publication of results (and therefore, the ability to have wider research impact). Unlike a laboratory, a zoo cannot breed or acquire the exact numbers of animals they need on demand for specific research purposes (i.e., produce the exact number of animals to find a significant effect in the data according to a statistical power calculation [Serdar et al. 2021]), and so it is important to explore opportunities for expanding small sample sizes in zoos.

Although there are benefits of opportunistic, “case study” styles which are often seen in zoo research, particularly when there is little known about a species or a topic, advancement of the discipline on a wider scale is only possible with larger sample sizes that are more reflective of the true population. Larger sample sizes also reduce the variability in effect sizes. Meta‐analyses have attempted to combine evidence across several small‐N studies, but this is an intensive process that requires complex statistical techniques, particularly when studies have used different methods or have vastly different sample populations. Multi‐institution research which increases N and improves generalizability to a wider context (e.g., to the species at large) is important, in particular, diversifying study populations, alongside acknowledging potential sampling bias (Webster and Rutz 2020).

### 2.2 Standardized Research Methods

Another benefit of multi‐institution research is the opportunity to develop and use standardized methodologies. This may include using the same inclusion/exclusion criteria, apparatus designs, sampling methods, types of software and programs, and plans for data organization and analyses across all research sites. Having standardized methodologies means that data can be combined and analyzed together, thus avoiding the need for retrospective comparative work. If more than one researcher collects the data, standardized methods, reliability testing, and observer training can be undertaken to ensure comparability.

Standardized testing helps zoo research avoid the “replication crisis” from low repeatability that has been noted in other fields (Wiggins and Christopherson 2019). This issue is also perpetuated by scientific journals and granting organizations, preferring to publish novel findings rather than replications, and perhaps a deeper human desire to innovate. The result of which is potentially noncomparable research data and non‐validated methods. In contrast, a standardized protocol can be assessed for validity, at a minimum in terms of face/content validity (i.e., is the methodology asking what it should be to answer the questions it sets out to? E.g., if measuring behavior to understand an animal's experience, has the researcher given a logical reason for its inclusion?) and construct validity (i.e., are the measures assessing that which they were designed to measure? E.g., are there correlations (positive or negative) between different behaviors which indicate the same or different welfare experiences?) (Meagher 2009). A further step toward criterion or predictive validity (e.g., comparison to external, independent measures showing that the measurement can be used to successfully predict future scenarios, e.g., being able to predict how an animal will respond in a given situation based on prior information) would come as a result of a completely validated protocol.

Finally, standardized methods help researchers commit to their research plan, rather than changing on the basis of incoming results. We acknowledge that sometimes research plans have to change due to unforeseen circumstances like poor weather conditions or apparatus faults, but a benefit of multi‐institution research is that data from a mistrial (e.g., experimenter error, apparatus fault, technological error) can be excluded without harm to the study's value. This, in turn, increases the reliability and validity of the results of the study overall. Standardized methods, including a requirement to pre‐register plans, also reduce the risk of hypothesizing after the results are known (HARKing). This refers to creating hypotheses and predictions after the data are collected rather than beforehand, potentially leading to type 1 errors and research designs that have not fully tested all relevant parameters for that question (e.g., collecting data on the impact of environmental variables on animal behavior and enclosure use but only hypothesizing that sunshine would increase their time spent engaging in active behaviors in their outside space once this phenomenon was observed in the data). A similar issue might be failing to report the original hypotheses and predictions if they are not supported by the data. By “pre‐registering” (declaring in advance) the aims, hypotheses, and predictions of a multi‐institution study, all institutions can consent to supporting the plan and avoid HARKing (Baumgartner et al. 2023) (e.g., stating ahead of time the expectation to see increased engagement in outside areas during sunny weather and designing the research to consider all variables related to this). Similarly, by pre‐registering the plan for data analysis, analysis bias can be avoided. There are several ways that bias in analysis can occur, but selective reporting of results is one of the most common (Stefan and Schönbrodt 2023). This is when results are reported in such a way as to support a pre‐defined narrative (i.e., presenting only those results that support a positive engagement with enrichment rather than all results which may also demonstrate social issues caused by the enrichment). P‐hacking, another analysis bias, refers to inappropriate manipulation of the data and statistical analysis, in an attempt to find any significant results rather than the ones originally proposed or in an attempt to validate the researcher's pre‐defined (but not reported) narrative (e.g., if the researchers wish to prove the effectiveness of a piece of enrichment they have designed they may retest the results, without appropriate adjustments, until they find a desired significant effect). Having standardized methodologies is particularly pertinent in the advancement of open science, which aims to reduce publication of data that are analyzed “until something fits.”

## 3 Current Multi‐Institution Research in Zoos

Here, we evaluate the benefits and drawbacks of current and distinct categories of multi‐institution research. Note, in places we refer to “researcher” in the singular but acknowledge there may be multiple people.

### 3.1 External Research

On a relatively small scale, ad hoc research projects run by an external (e.g., university) researcher can take place across a handful of zoos, with one or more species. The project is usually designed by the researcher rather than “in house” by zoos (although there may be overlapping interests), and different zoos may have varying levels of input in project design (Schulz et al. 2022). The purpose of sampling multiple zoos is usually to increase sample size, but there can be difficulties in successful execution. Professional zoo associations such as the British and Irish Association of Zoos and Aquariums (BIAZA) or taxon advisory groups (TAGs) encourage members to participate in a small‐scale project by endorsing its design and aims, but it is not typically the association's role to coordinate or run the research. Ad hoc research of this kind may be biased toward zoos which are local to the researcher's home institution (e.g., Bartlett et al. 2023; Gupta et al. 2019; O'Connor and Vonk 2022; Williams et al. 2020) and often focuses on a single species/task (e.g., Asian elephants, *Elephas maximus* [Barrett and Benson‐Amram 2020, 2021; Jacobson et al. 2022]). Data are gathered by the researcher who is limited by the resources available to them (Alligood et al. 2017; Shepherdson and Wielebnowski 2015).

While there are several examples of proactive collaborations between zoos and academia one potential issue with the majority of current multi‐institution zoo research has been project creation and leadership by a single external researcher (or research group) who collect data from multiple zoos based on questions that are driven by the external researcher (or groups) interest rather than zoos initiating and coordinating a multi‐institution project to ensure strategic goals of all stakeholders are met. This may be due to perceived and experienced practicality and feasibility (e.g., not all zoos have staff qualified or with sufficient time within their jobs to evaluate, develop, or lead studies), as well as the conventional view that zoos have a responsibility to provide universities or other educational institutions access to animals and other resources (Anderson et al. 2010).

### 3.2 Collaborative Research

On a slightly larger scale, a researcher may initially pose a research question but rely on their own network (across academia and the zoo/aquarium industry) to help perform the study in a more collaborative manner than above. As an example, several small‐scale multi‐institution projects arose opportunistically as a result of the COVID‐19 pandemic, comparing the responses of various species to zoo closures and reopenings (e.g., Frost et al. 2022; Kidd et al. 2022; Podturkin 2022; Williams et al. 2021, 2023). This demonstrates the ability of researchers and zoo staff to conduct research using a standardized protocol. Collaborative research may be instigated by researchers interested in the topic, single zoos wanting to extend an in‐house study, or wider groups (e.g., animal TAGs, veterinarians, or nutrition groups) who have identified research priorities (e.g., Dierenfeld 2005; Mylniczenko et al. 2019; Strong et al. 2018). Small‐scale collaborative research may also arise from industry‐led conferences or other networking events (e.g., AWRN 2024; Fernandes et al. 2019).

Larger‐scale collaborative projects may be motivated by an emerging welfare or conservation concern. These much larger projects may be coordinated or supported by a zoo association if the project is of considerable interest or value to its membership. Alternatively, the project may not be endorsed by a zoo association per se, but it may focus on zoos within one zoo‐accrediting association or geographic area (e.g., Europe or North America) for practical reasons. For example, large‐scale geographically isolated welfare projects (up to 40 zoos) have been instigated on elephants (*Loxodonta africana* and *E. maximus*) in Britain/Ireland (Yon et al. 2019) and North America (independent of each other) (Meehan et al. 2016) and polar bears (*Ursus maximus*) in North America (Shepherdson et al. 2013). Other types of collaborative research have leveraged veterinary samples and biobanking and cryobanking repositories of animal and plant materials (e.g., The FrozenZoo, NatureSafe, and EAZA Biobank‐Hildebrandt et al. 2021; Mooney et al. 2023). Unfortunately, these types of research often rely on having pre‐established networks and significant financial support and may therefore exclude researchers who are students/temporary researchers, more junior staff, early career researchers, and/or those working in different geographical areas, leading to a dominance of Western, Educated, Industrialized, Rich and Democratic (WEIRD) driven research (Henrich et al. 2010).

#### 3.2.1 Technology in Support of Collaborative Research in Zoos

Technological advances have facilitated multi‐institutional research and retrospective studies. For example, web‐based global species record‐keeping systems allow historic data to be input, stored, and accessed rapidly and securely (e.g., da Silva et al. 2019; Rendle et al. 2020; Rich et al. 2023). Although expansive and long‐term data sets are advantageous, the type of usable data on these systems may be limited; animal record‐keeping systems predominantly focus on demographic or other specific numerical data (i.e., weights, heights, birth and death rates, etc.). Species360, one of the largest zoo industry database providers, reports that they have more than 10 million records available for historical and live animals of over 21,000 species (Species360 2020). Sometimes, however, the membership requirements for large databases present a barrier to open research. Researchers can apply to access historically collected data. However, the information may be data protected or the research question may not be able to be answered with retrospective data. Furthermore, behavioral data are often stored alongside demographic data in an ad hoc manner, rather than in a systematic manner that would allow inter‐institutional comparisons (Wark 2024: https://community.zoomonitor.org). Historical data sets are imperfect because current researchers have limited knowledge or context about past methods used to collect data, and it is not always possible to retrospectively conduct inter‐rater reliability analysis. Finally, although there are significant benefits to using large data sets, they often require substantial manipulation to clean or deal with missing data points (Cole et al. 2012).

Where data are appropriate, researchers can systematically conduct meta‐analyses to answer new questions. New advances in computer software can allow behavioral data to be collected collaboratively and collated for analysis in one place (e.g., Wark 2022, 2024) or even analyzed from collated video footage through the use of artificial intelligence (Gübert et al. 2023), but so far while this is a successful data collection tool, it does not offer the option to collaborate on the planning and analysis, which is a key component of the ManyZoos model.

## 4 Introducing ManyZoos: A New Collaborative Approach to Multi‐Institution Zoo Research

### 4.1 An Introduction to ManyZoos

The concept of “Big Team Science” involves large‐scale global collaborations of researchers and other stakeholders working together to select a question and generate large and complex data sets to attempt to answer it. This process has grown in recent years (Coles et al. 2022; Stokols et al. 2008), driven in part by improvements in global communication systems (Coles et al. 2022; Forscher et al. 2023; Wu et al. 2019). Open science principles, including pre‐registration and openly sharing preprints and data, allow Big Team collaborations to increase transparency and reproducibility while preventing issues such as HARKing and P‐hacking (Chambers and Tzavella 2022). Another draw of Big Team Science is the ability to amass large sample sizes under standardized protocols, allowing new theories to be tested and modeled for advancement in the field (Alessandroni et al. 2023, 2024; Poo et al. 2022; Wu et al. 2019). The aim of the ManyZoos project (hereafter ManyZoos) is to facilitate a collaborative research network that works to advance the field of zoo science. The ManyZoos model follows that of the other “ManyX” initiatives born from the field of psychology that are forms of Big Team Science (e.g., Table 1). However, ManyZoos differs from these earlier initiatives owing to its lack of specificity in relation to the study species, focusing on broader subject areas with the opportunity to specialize in a range of species, and thus providing an opportunity to advance all aspects of zoo science.

**Table 1:** An example of some of the current Many initiatives, including their key aims/objectives and examples of projects which have arisen from these collaborative approaches.

Initiative	Aim/objective	Concept paper	Website
ManyBabies	Collaborative research practices that allow teams to test hypotheses about infant development	Frank et al. (2017)	https://manybabies.org
ManyBirds	Aims to provide new insight into the evolution of avian cognition and behavior through multi‐site comparative studies and large‐scale collaboration	Lambert et al. (2022)	https://themanybirds.com
ManyDogs	An international consortium of researchers interested in taking a big team science approach to understanding canine behavioral science	Alberghina et al. (2023)	https://manydogsproject.github.io
ManyFishes	Collaboration across researchers and institutions to increase both the number and diversity of fish samples used in cognitive research	N/A	https://themanyfishes.github.io
ManyGoats	A collaborative international team of experts aiming to improve the external validity and dissemination of our findings in the study of goat behavior and welfare by implementing identical experimental protocols to simultaneously test goats across many different facilities around the world	ManyGoats et al. (2024)	https://www.themanygoatsproject.com
ManyManys	Collaborative group developing and applying innovative methods to measure and compare behavior across animal taxa	Alessandroni et al. (2023)	https://manymanys.github.io
ManyPrimates	Initiated to facilitate collaboration across study sites in primate cognition research	Many Primates et al. (2019)	https://manyprimates.github.io

ManyZoos is an initiative designed to facilitate cross‐industry collaborative research between terrestrial zoos and aquaria (hereafter “zoos”) and academic partners. This open science initiative is designed to support the development of high‐quality collaborative research which addresses key questions and furthers knowledge in the field of evidence‐based science in zoological collections. The aim of ManyZoos is to create an accessible infrastructure and collaborative network which will support the development of individual projects or project methodologies using standardized methods for undertaking repeatable research in zoological collections (hereafter zoos). By providing the opportunity for unrestricted collaboration, ManyZoos will enable the development over time of big data sets which can be used to enhance practical and theoretical knowledge in relation to animals in zoos and the work of zoos.

The overarching ManyZoos initiative will consist of a number of committees which support aspects which are relevant to any ManyZoos research project (Table 2). These committees will have supported development of the ManyZoos network through the creation of standard operating procedures for ManyZoos, codes of conduct for members, and ethical protocols for prospective research projects.

**Table 2:** An overview of the ManyZoos project committees.

ManyZoos committees	Aims of the committee
Communications and outreach	Communication, both internally with the members and externally with academic and non‐academic (i.e., zoo/aquariums) professionals.
Animal welfare and ethics	Support development of ethical and welfare‐friendly research projects. Develop guidelines and criteria for fair and transparent contributionship.
Data and technology	Develop and support data management and reproducibility standards for ManyZoos research projects. Support ManyZoos technical infrastructure.

**Figure 1:**
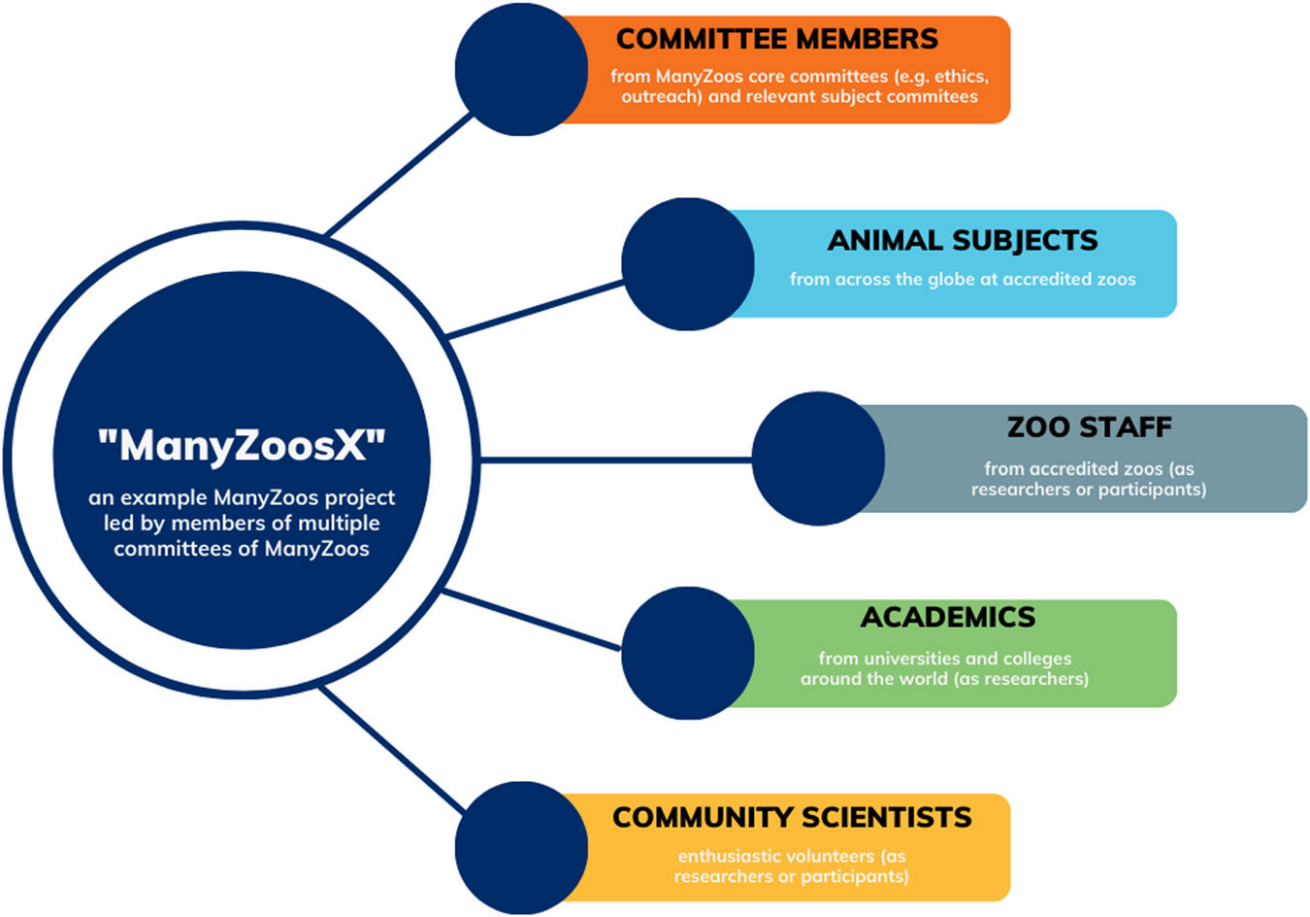
The stakeholders and resources required for a hypothetical ManyZoos project “X.”

**Figure 2:**
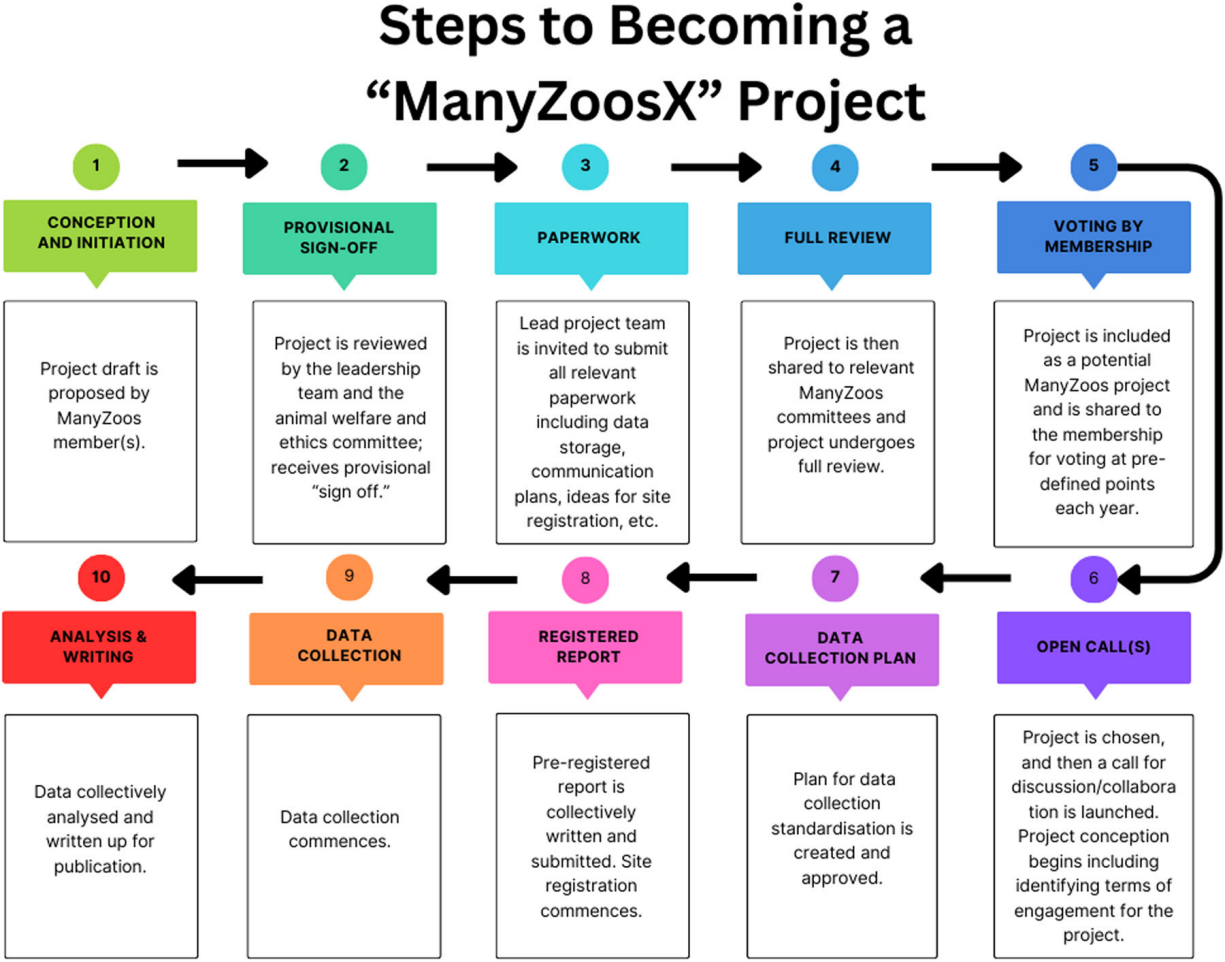
Steps for project inclusion as a “ManyZoosX” project.

The infrastructure of an example of a specific ManyZoos project is displayed in Figure 1. A particular project (e.g., ManyZoos1) will consist of a lead project team who conceives the project and presents the project for consideration by the ManyZoos leadership team and committees. Once the project is identified as suitable, this project team would then be in charge of planning logistics, developing protocols for handling and analyzing data, and lead on producing and disseminating results. This team may be zoo staff or volunteers, academics, government agency workers, and so forth. In addition to the lead project team, there would be any persons who wish to join the project and contribute to its development and execution. A ManyZoos research project would then be developed from the ground up as per Figure 2.

Zoological collections have access to standardized data collection software systems (e.g., ZooMonitor, Wark et al. 2019; Wark 2022, 2024). The aim of ManyZoos is not to replace any of the existing approaches to multi‐institution research. Rather, ManyZoos addresses the current need for collaborative, open science in zoos, leveraging the technology and data storage systems which are readily available and used widely by zoological collections in day‐to‐day management.

### 4.2 Collaboration & Networking

ManyZoos aims to strengthen connections within the zoological/aquarium community and the wider scientific community. This is important to us as we are passionate about uniting perspectives from diverse disciplines and taxa in an effort to better understand species and improve management for animals in zoological collections. While zoo staff undoubtedly bring a range of expertise, many of which is taxon‐ or species‐specific, smaller zoos may not have staff with expertise for all elements of a project, particularly if it is multidisciplinary. ManyZoos facilitates connectivity that would otherwise not exist, and it recognizes and celebrates expertise across its network. With a large network of contributors, ManyZoos will enable zoo staff to actively participate in research that they may not have had time or resources to lead on their own. And, by developing standardized methods that have been tested, published, and posted online, the ManyZoos network can contribute tools for zoos wanting to do research in the future to take on a more manageable (i.e., previously evaluated) research project that will directly inform decision‐making at that zoo. Sharing of knowledge through open science practices will enable those not directly involved in the research to benefit from the knowledge generated through the completion of ManyZoos projects. In other words, zoos do not need to contribute to a “ManyZoos Project” to benefit from the products of the consortium.

### 4.3 Data Collection, Ownership, and Management

When thinking about the value of zoos for research, a ManyZoos collaborative approach optimally leverages zoos and their resources for the betterment of science and ultimately, animal wellbeing.

i. Data collection
  Zoos and aquariums are unique, and the aim of ManyZoos is to utilize this to the advantage of research teams, to increase the generalizability of findings, rather than zoos or researchers working in silos without the opportunity for increased sample sizes and collaborative approaches to data collection. Although Big Team Science has many benefits, we acknowledge the limitations; variations within animal environments, husbandry, and individuality cannot be controlled for but by following the STRANGE framework (Webster and Rutz 2020), these variations can be accounted for within a large data set. Additionally, the complication of a large team of data collectors can be a drawback (McAlearney et al. 2023). There may be concerns, for instance, about data trustworthiness, and coders and analyzers may be needed, especially when we consider the qualitative data on animal or zoo visitor behavior. Standardization training (e.g., inter‐rater reliability training and building projects with practitioners) and the use of coding apps can assist in overcoming some of these issues (e.g., Van Der Marel et al. 2022; Wark et al. 2021). This is incorporated into the code of practice for developing ManyZoos research projects. Difficulties associated with working in large groups will be overcome through the ManyZoos code of conduct and having clear working practices for ManyZoos research projects defined at the onset of research projects. Each ManyZoos research project will be required to develop their own working practices to ensure the smooth running of projects, with a clear strategy for decision making and conflict resolution to be laid out at the beginning of the project.
ii. Data ownership, storage, and open access repositories
  Secure data storage and protocols for ensuring data are used appropriately are paramount for ManyZoos to work without risks to participating zoos. It is vital to strike a balance between open science practices and protecting the confidential data of zoos and other institutions. To ensure zoo data are protected and rigorous processes are followed, persons creating the project will need to be approved by the ManyZoos leadership team and relevant committees and have signed the ManyZoos code of conduct. To propose a project, lead project teams must have a project plan which includes clear information on data storage. Those contributing data, that is, zoos or the zoos representative (e.g., researchers within zoos), will be responsible for collecting data. Data will be pseudo‐anonymized (i.e., the zoo will not be identifiable but data from each facility will still be linked) by the persons collecting the data before submitting it to the lead project team. Data for the project will then be stored by the lead project team at their home institution, and that data will only be used for the project for which zoos and/or their representatives have signed up. Only completely anonymized, consented data will be available in open access repositories, and all persons contributing data must sign up to a data use policy before submitting data. All persons contributing data remain the owners of that data and can use the data as they wish for future work. All activities will be guided by a pre‐defined code of conduct to ensure openness and inclusion should not come at the expense of individual or institutional safety.

### 4.4 Publishing a ManyZoos Project

As per open science frameworks, all ManyZoos papers will be published wherever possible in open‐access formats with registered reports made available where suitable. ManyZoos will use a co‐authorship agreement (based on the CRediT taxonomy) which all persons (including zoo staff members) contributing to a research project must agree to at the onset of the project, and all contributors will have the opportunity to review manuscripts before publication.

## 5 Future Directions for ManyZoos

ManyZoos is a new approach and is likely to be a slow evolution rather than a rapid revolution. ManyZoos will be defined and refined by its membership over the coming years, in the same way other ManyX initiatives have been shaped by their own communities, including a proof‐of‐concept study to allow for a test of our network and organization structure, before executing more complex ideas (Baumgartner et al. 2023). We are developing partnerships with organizations such as Minorities in Aquarium and Zoo Science (MIAZS 2024) and the Animal Behavior Management Alliance (ABMA 2024) to help increase the accessibility of zoo research. By acting on the evidence that prioritizes the best direction for zoo research and collaborating to determine the next steps for the ManyZoos research focus, we can work together to advance our knowledge of conservation and zoo management. At the time of writing, the first ManyZoos project (ManyZoos 1) has been launched and is currently open for contributors. The idea of this project is to test the efficacy of the network and its processes. Colleagues are encouraged to join the free ManyZoos collaboration, join the ManyZoos 1 project, and submit project proposals for consideration for the ManyZoos 2 project.

## 6 Conclusion

We are currently experiencing the world's sixth mass extinction event, and as such, there has never been a more pressing need for collaboration and research to aid species conservation (Clark et al. 2023). Zoos have a clear role in modern society with zoos sitting in the middle of a number of conservation and societal activities (Spooner et al. 2023). Traditionally, the work of zoos has centered on four pillars (education, conservation, research, and recreation), with animal welfare embedded within these (Reade and Waran 1996). Evidence‐based practice is paramount in the advancement of the work of zoos. ManyZoos seeks to provide opportunities for collaboration within the zoological industry, with the aim of supporting the development of large and complex data sets, which can be used to answer a plethora of questions within zoo science. In doing so, the ManyZoos initiative will support more robust, evidence‐based decision‐making for zoo and aquarium animal management and conservation.

## Data Availability

Data sharing is not applicable to this article as no data sets were generated or analyzed during the current study.
